# Physick’s Forceps

**Published:** 1850-04

**Authors:** A. Berry


For the Dental Register of the West

### PHYSICK'S FORCEPS.

BY A. BERRY, D. D. S.

If Physick's forceps for the extraction of the dentes sapientiae were fairly tried by our profession generally, it would, in my humble opinion, be much more highly appreciated than at present. Dr. Keocker is, so far as my information extends, the only dental author who notices them; which he does as follows:

"For removing the dentes sapientiae, and sometimes the second molares, I have occasionally used an instrument invented by Dr. Physick, of Philadelphia, of which I beg to give a short description.

"This instrument is in the form of a strong pair of tooth forceps; of which the parts which commonly form the claws are two blunt blades, somewhat in shape like those of a pair of large nail scissors, and in an oblique direction.

The tooth is removed by placing the two claws between the tooth to be extracted and its anterior neighbor, with sufficient pressure to force the tooth towards the posterior part of the mouth, in order to destroy the periosteum; the tooth is then to be lifted out of its socket by the same instrument or with another pair of forceps.

In those cases where the anterior teeth are sound and firmly seated in the sockets, and when the anterior part of the tooth to be removed is not too much destroyed by decay or caries, I have found this instrument very well adapted for the operation."

Every dentist knows the difficulty frequently experienced in the removal of the inferior dentes sapientiae. When they are firmly seated in strong alveoli, as their fangs often incline very much towards the caronoid processes, it sometimes requires considerable force as well as tact to extract them with the forceps commonly made for the purpose. It seldom happens in such cases that a little force applied between these teeth and the second molares, if remaining, fails to elevate them, when they may easily be lifted out of their sockets. An elevator is sometimes used for this purpose, but no one, I think, who has ever employed Physick's forceps in a case of this kind would ever abandon them for any other instrument for luxating the dentes sapientiae.

Their blades should be wedge shaped and sharp pointed, so that if the tooth to be extracted, whether superior or inferior dens sapientiæ, has lost most of its crown by decay, they may be inserted beneath the gums so as to luxate its fangs when closed.

This instrument will also answer very well to divide the molares, when it is requisite, for the purpose of removing their fangs separately.

A dentist called at our office in Cincinnati a few years ago with a pair of these forceps, which he proposed to sell to me for the trivial sum of five dollars, *asserting they were his own invention.* Those excellent cutlers, Mr. Sherwood and Mr. Rees of Cincinnati, manufacture them of the best finish for two dollars; and if our ingenious confrere, who was then making a tour, could have sold a pair to each of the forty dentists then in the city, the profit might have been of material assistance in pursuing his journey.

